# Effect of Palmitoylethanolamide Compared to a Placebo on the Gut Microbiome and Biochemistry in an Overweight Adult Population: A Randomised, Placebo Controlled, Double-Blind Study

**DOI:** 10.3390/biomedicines12071620

**Published:** 2024-07-20

**Authors:** Romeo Batacan, David Briskey, Yadav Sharma Bajagai, Chelsie Smith, Dana Stanley, Amanda Rao

**Affiliations:** 1 School of Health, Medical and Applied Sciences, Central Queensland University, Rockhampton, QLD 4701, Australiay.sharmabajagai@cqu.edu.au (Y.S.B.); d.stanley@cqu.edu.au (D.S.); 2 School of Human Movement and Nutrition Sciences, The University of Queensland, Brisbane, QLD 4006, Australia; d.briskey@uq.edu.au; 3 RDC Clinical, Brisbane, QLD 4006, Australia; chelsie@rdcglobal.com.au; 4 Institute for Molecular Bioscience, The University of Queensland, Brisbane, QLD 4006, Australia

**Keywords:** palmitoylethanolamide, microbiota, metagenomics, cytokines, NAD, smooth LPS

## Abstract

This study investigates the effects of palmitoylethanolamide (PEA) on the gut microbiome of overweight adults. Fifty-eight participants (twenty males, thirty-eight females) aged 18–65 years with a BMI range of 30–40 kg/m^2^ were recruited. Participants were randomised to receive PEA (*n* = 36) or a placebo (*n* = 22) for 12 weeks. Microbiota composition, richness, diversity, and metabolic functions, faecal short chain fatty acids and calprotectin, pathology markers, and health-related questionnaires were analysed throughout the 12 weeks of supplementation. PEA supplementation significantly reduced triglyceride levels and IL-2 concentrations. No significant differences were found in the overall microbiota composition between the groups, and microbiota richness and diversity remained consistent for both groups. Functional analysis demonstrated no differences in functional richness and diversity, but specific pathways were modified. PEA supplementation resulted in a decrease in the abundance of pathways related to aromatic compound degradation, NAD interconversion, and L-glutamate degradation, while pathways associated with molybdopterin biosynthesis and O-antigen building blocks exhibited increased abundance. Increased production of O-antigen results in smooth LPS associated with reduced pathogenic stealth and persistence. PEA supplementation may influence specific microbial species, metabolic pathways, and reduce serum triglyceride and IL-2 concentration, shedding light on the intricate relationship between PEA, the microbiome, and host health.

## 1. Introduction

Obesity has emerged as a global health concern, with increasing rates of occurrence observed in numerous countries worldwide. The worldwide prevalence of obesity in 2019 was reported to be 12.2% in men, and 15.7% in women, with the forecast that 1 billion adults will be obese by 2025 [1]. This complex disease is characterised by low-grade chronic inflammation, which predisposes individuals to a range of chronic conditions, including cardiovascular diseases, type 2 diabetes, and metabolic syndrome [2]. While multiple factors contribute to obesity, poor diet and inadequate physical activity, especially in the developing years, play a significant role in its development [3]. Managing obesity and its associated issues requires a comprehensive approach, incorporating lifestyle modifications such as dietary changes and increased physical activity, along with medical interventions like pharmacological therapy, surgical procedures, and the potential use of dietary supplements [4]. Dietary supplements are gaining popularity as an alternative to traditional therapies due to their low toxicity profile and widespread availability [4].

Recent studies have investigated the use of dietary supplements to mitigate inflammation associated with obesity [5,6]. The findings highlight the promising potential of dietary supplement interventions in reducing circulating inflammatory markers like TNF-α, IL-6, and CRP [5,7]. Some dietary supplements with recognised potential anti-inflammatory properties include Vitamin D, Coenzyme Q10, turmeric, quercetin, ginger, probiotics, and palmitoylethanolamide (PEA) [8,9,10,11]. PEA has gained interest in recent years for its unique anti-inflammatory mechanism of action. Unlike other supplements that primarily target specific receptors or enzymes, PEA exerts its anti-inflammatory effects by primarily activating peroxisome proliferator-activated receptor-alpha (PPAR-alpha) and indirectly modulating the endocannabinoid system [12]. PEA, in contrast to cannabidiol derived from the cannabis plant, shares a structural similarity to anandamide (AEA) or the “bliss molecule”, enabling PEA to enhance the effects of AEAs and inhibit their breakdown within the body [13].

PEA was first isolated in 1957 from soybean lecithin, identified as N-(2-hydroxyethyl)-palmitamide [14], and later found in many plants and animals. In the 1930s, studies found that feeding dried egg yolk, which also contains PEA, prevented rheumatic fever recurrence in susceptible children and protected guinea pigs against anaphylactic arthritis [15]; these observations highlighted the potential anti-inflammatory properties of PEA. Subsequent investigations confirmed the anti-inflammatory effects of the lipid fraction in egg yolk, and a similar compound was also purified from peanut oil and vegetable lecithin [16,17]. In the 1970s, numerous placebo-controlled double-blind clinical trials demonstrated the anti-inflammatory and immune-modulating properties of PEA in the treatment of influenza and the common cold [16]. Since then, PEA has been discovered in a wide range of plant and animal food sources, leading to an increasing interest in its functions within the mammalian body [18].

PEA levels in mammalian tissues are believed to increase during cellular stress and tissue injury, although the specific physiological stimuli regulating its levels remain unclear [19]. Studies have shown increased PEA accumulation post-mortem in the pig brain [20], as well as elevated levels in the ischemic brain [21] and in response to ultraviolet-B irradiation in mouse epidermal cells [22]. Adipose tissue, known for its involvement in systemic secretion of IL-6 and leptin, can secrete significant amounts of PEA [23]. Lipopolysaccharide (LPS) can influence human adipose tissue to modulate its inflammatory state [19,24]. LPS strongly inhibits the release of leptin from adipose cells, and PEA enhances this inhibitory effect. Interestingly, these actions are not associated with decreased leptin gene transcription [19]. Therefore, PEA does not have an anti-inflammatory role in IL-6 secretion via nuclear factor kappa B at the adipocyte level. Instead, it appears to play a role in the LPS-stimulated pathway, which independently inhibits leptin secretion [19].

A review of the research suggests dosage of PEA ranges from 300 mg to 1200 mg daily [25], while other evidence suggests a dose of 700 mg is effective at pain relief [26,27]. However, PEA is typically poorly absorbed; therefore, different formulations of PEA may have different efficacies for the same dose. The formulation used in this study (Levagen+; Gencor Pacific, Hong Kong) has previously been shown to have superior absorption over standard PEA [28].

The recognition of the link between LPS and PEA has led to the development of this study. It is proposed that the inhibition of LPS could potentially be attributed to alterations in the microbiome and modulation of the gastrointestinal mucosa. It is plausible that PEA functions by preventing the passage of LPS across the gut–blood barrier. Therefore, the aim of this study is to evaluate the effectiveness of PEA in altering the diversity and population of the gut microbiome and the subsequent effects on health markers compared to a placebo in overweight but otherwise healthy adults aged 18–65 years old.

## 2. Materials and Methods

### 2.1. Inclusion and Exclusion Criteria

This study was conducted in Brisbane, Australia, with participants enrolled between March and October 2021. This study had a 14-day screening period, with participants required to complete screening via telehealth and in clinic. A total of 58 participants (20 males and 38 females) were enrolled to participate in the study. Participants were included in the study if they were 18–65 years of age and had a body mass index (BMI) ranging from 30 to 40 kg/m^2^. Participants were excluded if they had history of unstable or severe illnesses, recent acute sickness, ongoing use of certain medications or gut-altering supplements, active smoking, chronic alcohol use, allergies to study ingredients, pregnancy or lactation, females of childbearing potential (i.e., not using a form of contraception), and medical prescriptions affecting the immune and/or inflammatory response. Diabetes and other metabolic diseases were only permitted if they were stable (no change in medication for > 3-months) and not considered severe. Acceptable forms of highly effective contraception include established use of oral, injected or implanted hormonal methods of contraception, placement of an intrauterine device (IUD) or intrauterine system (IUS), sterilised male partner, and true abstinence. Condoms, periodic abstinence, withdrawal and spermicides were not acceptable methods of contraception. Additionally, participants with conditions deemed unsuitable by the investigator, recent participation in related studies, or a history of cancer, HIV, or chronic steroid use were not eligible for the study. All participants were asked to maintain their usual diet and exercise habits for the duration of the study. Eligible participants that met the study criteria provided written informed consent and were randomised into the study. 

### 2.2. Study Groups

This study was divided into two groups, with the participants and investigators being blinded to each group. Participants were randomly assigned to groups in a 2:1 ratio, using Random Allocation Software (https://www.sealedenvelope.com/simple-randomiser/v1/new, accessed on 10 July 2024). Group A (*n* = 36) received an active treatment of PEA (Levagen+). PEA is a Therapeutic-Goods-Administration-approved ingredient for use in listed medicines in Australia (Brand name Levagen+; Ingredient name palmidrol, ID 105628). PEA was provided by Gencor Pacific (Hong Kong) and encapsulated in a TGA facility to good manufacturing practice (GMP) conditions. PEA (350 mg per capsule) was taken daily, one in the morning and one in the evening with water. 

Group B (*n* = 22) received a placebo consisting of microcrystalline cellulose (MCC) encapsulated in an opaque capsule, appearing visually identical to the PEA product. The placebo was provided by a Brisbane-based compounding Pharmacy, manufactured in compliance with GMP requirements. The placebo (300 mg per capsule) was also administered daily, one in the morning and one in the evening. 

### 2.3. Study Protocol

The study was conducted in compliance with the current International Conference on Harmonization (ICH) Guideline for Good Clinical Practice (GCP), the Therapeutic Goods Administration (TGA) Note for Guidance on Good Clinical Practice, and the ethical guidelines outlined in Additional Ethical Considerations. The study followed a double-blind, placebo-controlled, randomised, single center study design. Participants were recruited through databases and public media outlets. Interested individuals were screened against the study criteria and received a detailed explanation of the trial and its requirements. Once deemed eligible, participants were asked to provide electronic, written consent (WP E-Signature—Versions 1.5.6.9 to 1.8.8, Phoenix, AZ, USA) to enroll in the study.

Once enrolled in the study, participants were randomly allocated to either the placebo or PEA group. Each participant received an opaque bottle containing capsules, with all bottles and capsules for both groups appearing identical. 

Following consent, and prior to starting on the trial product, participants were required to provide a number of baseline measures. Participants were required to provide a blood sample, a liver scan (fibroscan), basic anthropometric measures (height, weight, hip and waist circumference), a faecal sample, and completed questionnaires [24 h dietary recall, SF-36 (quality of life), the Perceived Stress Scale (PSS), and the Pittsburgh Sleep Quality Index 10]. Questionnaires were all completed electronically, using the participant identification code as the identifier.

Faecal sample collection was conducted by the participant in the privacy of their home and returned to the clinic within 7 days of collection. Participants were provided with a faecal collection kit (OMR-200; DNA Genotek, Stittsville, ON, Canada) and instructions during their first clinic visit. Once returned to the clinic, faecal samples were stored at −80 °C until analysis. Faecal samples were analysed for microbiome quantification, metagenomics, calprotectin, and short chain fatty acids (SCFAs).

All blood samples were collected from the antecubital fossa into a serum collection and EDTA collection tubes (BD, Sydney, NSW, Australia). Once collected, EDTA was immediately centrifuged (4 °C at 2100× *g* for 10 min), and serum was allowed to clot for 30 min before centrifugation. Once separated, plasma and serum aliquots were stored at −80 °C until analysis. Serum or plasma were analysed for enzyme and liver function tests (E/LFT), occludin, Muc2, endotoxin, GLP-1, GST, glutathione, FABP, homocysteine, IFN-g, TNF-a, IL-2, MCP-2, IL-1b, TGF-b, and high-sensitivity CRP.

Once all baseline measures were completed, participants began consuming the allocated study product for 12 weeks. Participants were contacted by telehealth consult throughout the 12-week study to maintain enrolment, ensure study compliance, and monitor for potential adverse events. Throughout the 12-week study period, participants underwent the same testing as the baseline at week 6 (mid-point) and week 12 (endpoint). At the week-12 clinic visit, participants were required to return all unused trial products for compliance monitoring.

### 2.4. Statistical Analysis

Twenty-two participants were required per group for statistical power to detect a change of 20% in an individual microbiome species (e.g., *Akkermansia muciniphila* effect size: 0.55; alpha error probability: 0.05; power: 0.8). Up to 40 participants were recruited to each group to allow for a predicted 45% dropout rate. Statistical analysis was conducted by first testing for normality within each dataset. Based on normality, the appropriate test was selected for analysis (i.e., *t*-test for normalised data or Wilcoxon for non-normalised data) of the two groups.

### 2.5. Biochemistry

E/LFT and hs-CRP were analysed on a Biobase BK-200 analyser using kits and calibrators from Biobase (Jinan, China). Occludin, Muc2, endotoxin, GLP-1, GST, glutathione, FABP, homocysteine, IFN-g, TNF-a, IL-2, MCP-2, IL-1b, and TGF-b were analysed using validated ELISA kits and calibrators from the local distributor (Jomar Life Research, Mulgrave, VIC, Australia).

### 2.6. Shotgun Metagenomic Sequencing

The DNA from the faecal sample was isolated using DNA mini spin columns (Enzymax LLC, Lexington, KY, USA), and quality was assessed with Qubit3.0 (Thermo Fisher, Waltham, MA, USA) and agarose gel electrophoresis. Covaris S220 (Covaris, Woburn, MA, USA) was used to fragment the DNA, followed by end repair, dA-tailing, adaptor ligation, and purification. The purified DNA then underwent size selection, followed by PCR amplification and library construction. The concentration of the resulting library was measured with Agilent 2100 (Agilent, Santa Clara, CA, USA) and qPCR before sequencing using Illumina Novaseq6000 with 150PE configuration (San Diego, CA, USA). The number of sequences in raw sequencing data was 38.24 ± 3.5 million (mean ± SD) per sample. 

### 2.7. Sequence Quality Control and Preprocessing

Data integrity was verified with Message-Digest-Algorithm-5-generated cryptographic hash [29]. The quality of each sequence file was analysed with fastQC v0.11.2 [30], and individual quality analysis files were merged with multiQC v1.11 [31] to visualise the overall quality of the sequences. The fastp tool was used for initial quality filtering and trimming to remove reads with too-low quality and too-short length (<50 bp). 

Any remaining sequencing adaptors in sequence reads were also trimmed with fastp. Further quality trimming of the data was undertaken with KneadData v0.7.10, applying Trimmomatic [32], filtering leading and trailing bases with a quality score of less than three and using a sliding window threshold of 4:15. The host DNA contamination was removed by aligning the sequences with the human genome. The number of sequences after quality filtering and host DNA decontamination was 36.97 ± 3.4 million (mean ± SD) per sample with a minimum Phred score of 35 and less than 1% overrepresented sequences in each sample. 

### 2.8. Taxonomic and Functional Analysis 

The trimmed and clean sequences were analysed for microbial taxonomic profile and function using the HUMAnN v3.6 [33] with UniRef90 [34] databases for profiling molecular functions and metabolic pathways. The downstream statistical analysis and visualisation were undertaken with the R program using packages Phyloseq [35], Vegan [36], and Microeco [37].

### 2.9. Faecal SCFAs and Calprotectin

SCFAs were analysed using a method based on a previously published assay [38]. The sample was first treated with sulphuric acid and then extracted into ethyl ether for analysis. Samples were separated and detected using a Shimadzu GC-FID. 

Faecal calprotectin samples were dehydrated using a centrifugal vacuum dryer, and dry weights were calculated. The samples were reconstituted in assay buffer and calprotectin measured as per instruction using an ELISA kit (Epitope Diagnostics Inc., San Diego, CA, USA). The plates were read on a Biobase BL-EK-10C plate reader (Jinan, China) as per the kit manufacturer’s recommendations.

### 2.10. Fibroscan

Liver scans (fibroscan) were undertaken at a local imaging center (I-MED Radiology, Brisbane, QLD, Australia) by a trained radiographer. Each fibroscan measured the shear wave velocity of the liver to determine liver stiffness and the presence of any abnormalities or liver steatosis.

## 3. Results

### 3.1. Demographics

Fifty-eight participants (age range 23–64 years) were enrolled in this study, with forty-four participants completing the full study requirements. Of the 14 participants (Group A = 7; Group B = 7) that failed to complete the study duration, 3 withdrew prior to starting on product, 1 had cirrhosis detected at the baseline liver scan, 2 were lost to follow up, 1 withdrew for personal reasons, 2 started antibiotics for URTI’s, 2 developed colds, 1 was not compliant, and 2 withdrew from adverse events. A total of six (four in Group A; two in Group B) adverse events were reported during the study period. In Group A, one person reported nausea following consumption of the trial product, one reported stomach cramps and upper back pain consistent with gallbladder issues they had previously experienced, one reported loose stools, and one reported restless sleep. In Group B, one reported their period not stopping and suspected they were peri-menopausal, and one was found to have kidney stones. Of the adverse events reported, three in Group A (nausea, loose stools, and restless sleep) and none in Group B were considered to be possibly product related as determined by the medical doctor.

Of the 44 completed participants (Group A = 29; Group B = 15), 40 participants (Group A = 27; Group B = 13) provided both a baseline and final blood sample. Forty-four participants provided stool samples, with thirty-one (Group A = 19; Group B = 12) being viable for analysis. There were no significant differences between groups for any baseline measures (Table 1); however, diastolic BP trended towards significance for change from baseline (*p* = 0.08). There were no significant differences between groups for any 24 h diet recall measures (Table 2).

### 3.2. Biochemistry

There were significant differences between groups for change from baseline in triglycerides and IL2 (Table 3; *p* < 0.05). No other significant differences in biochemistry markers were observed between groups or at different time points (Table 3).

### 3.3. Taxonomy

#### Microbiota Profile

**Figure 1:**
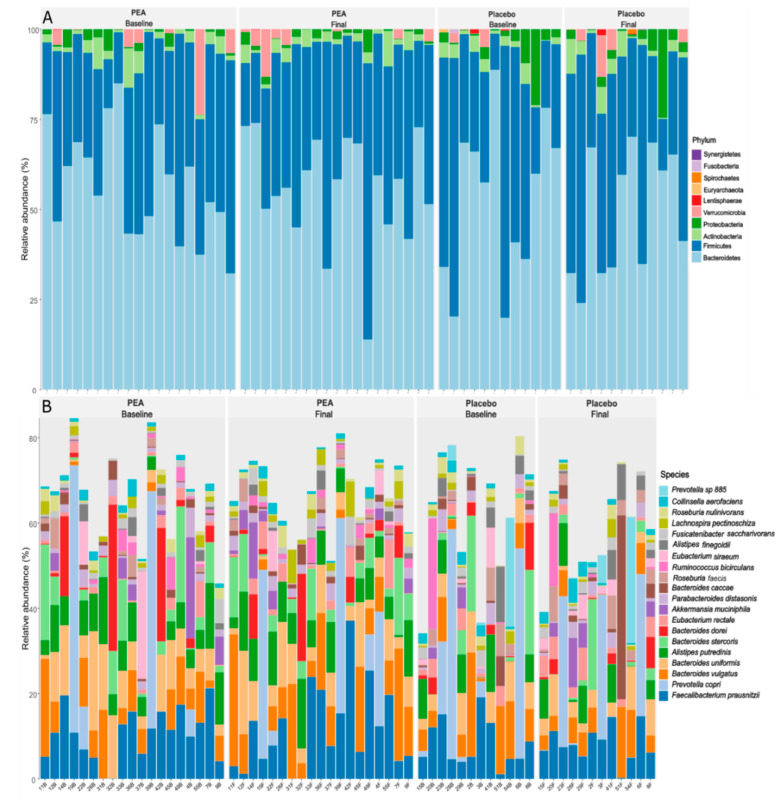
Relative abundances: The relative abundances of the top 10 phyla (**A**) and top 20 species (**B**) in individual participants in the two groups. At the phylum level, the individual participant has either Bacteroidetes or Firmicutes as a dominant phylum (**A**); while at the species level, the dominant species are either *Faecalibacterium prausnitzii* or *Prevotella copri* or *Bacteroides vulgatus* or *Bacteroides uniformis* or *Alistipes putredinis* or *Bacteroides stercoris* or *Bacteroides dorei* (**B**).

The top 10 phyla of microbes for Group A and Group B baseline and final measurements were Bacteroidetes, Firmicutes, Actinobacteria, Proteobacteria, Verrucomicrobia, Lentisphaerae, Spirochaetes, Euryarchaeota, Fusobacteria, and Synergistetes. Bacteroidetes and Firmicutes constitute ~90% of the total microbiota (Figure 1A). At the genus level, *Bacteroides*, *Prevotella*, *Faecalibacterium*, *Alistipes*, *Roseburia*, *Parabacteroides*, *Eubacterium*, *Ruminococcus*, *Akkermansia*, and *Oscillibacter* represented the 10 most abundant genera. At the species level, the top 20 microbial species for all groups are identified in Figure 1B, which constitute around 60–65% of total microbial species. There were no significant differences in microbiota composition between groups as tested with PERMANOVA based on Bray–Curtis and Jaccard distances between samples. However, there was a significant influence of gender on microbiota composition (PERMANOVA, *p* value < 0.01). While gender significantly influenced microbiota, based on the insignificant interaction between gender and treatment (*p* = 0.989), gender did not influence the findings on the effect of treatment on microbiota.

### 3.4. Alpha Diversity and Microbiota Richness 

**Figure 2:**
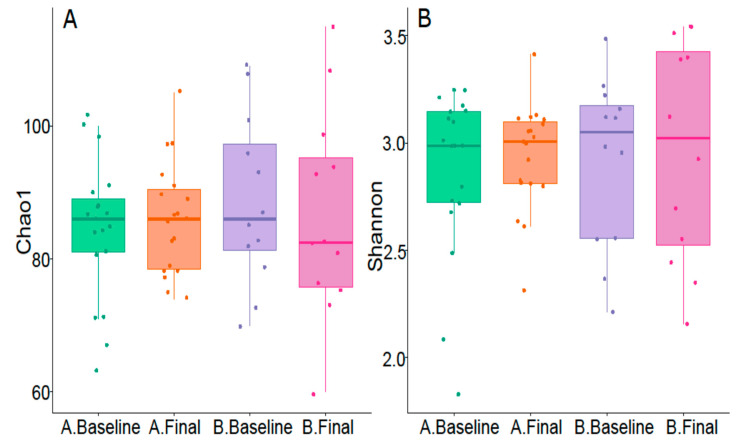
Alpha diversity and richness variation of the two groups: (**A**) = Chao1 index; (**B**) = Shannon index. A.Baseline = PEA baseline; A.Final = PEA final; B.Baseline = placebo baseline; B.Final = placebo final.

There were no differences in alpha diversity and microbiota richness between groups as analysed with the Shannon diversity index and Chao1 index (Figure 2).

### 3.5. Beta Diversity and Overall Microbiota Profile

There were no differences in beta diversity measured based on Bray–Curtis and Jaccard distances between the baseline and final in both Group A and Group B. Similarly, within group sample-to-sample distances were also similar between baseline and final in both groups. 

### 3.6. Differential Taxa

Although there were no significant differences in the overall microbiota profile between baseline and final measurement on both groups based on multivariate analysis, Metastat analysis revealed that the relative abundances of several microbial species were significantly different.

None of these differential species were in the dominant species list (top 20). The complete list of differential species is given in the Supplementary Tables S1 and S2.

### 3.7. Microbial Metabolic Functions

#### Composition

**Figure 3:**
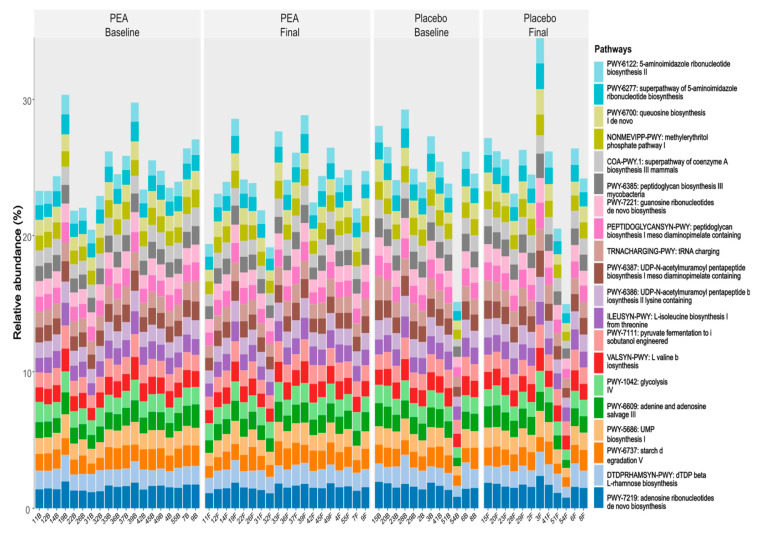
Relative abundances: the relative abundance of the top 20 metabolic pathways in all participants in the two groups (PEA and placebo) at baseline and final sampling points.

The top 20 microbial metabolic pathways (MetaCyc database) for Group A and Group B at baseline and the final sampling points are shown in Figure 3. The top 20 pathways represent about 25% of all metabolic pathways. 

### 3.8. Functional Diversity

The microbial functional diversity and richness were analysed at the gene ontology (GO) level by grouping the functional genes into GOs. There were no differences in functional richness and alpha and beta diversity between baseline and final measurements in Group A and Group B.

### 3.9. Differential Pathways

Although there were no significant differences in the overall functional profile between baseline and final measurement in both groups, Metastat revealed that relative abundances of 12 metabolic pathways were significantly different between the baseline and final sampling time points of the PEA group (Group A), as shown in Table 4. There were no significantly different pathways between the baseline and the final timepoint in the placebo group (Group B). The pathway PWY.6167—flavin biosynthesis II (archaea)—was the only difference between A and B at the baseline, significantly higher in B. There were no significant differences between the other relevant comparisons. 

### 3.10. SCFAs and Calprotectin

SCFA results showed that there were no significant differences seen between groups or between baseline and week-12 data (Table 5). The change from baseline for hexanoic acid in terms of the percentage of SCFA trended towards significance (*p* = 0.06).

There were no significant differences between groups or between baseline and week-12 values from the liver safety scans (Table 6).

### 3.11. Questionnaires 

There were no significant differences seen between groups or between baseline and week-12 values for the results from the SF-36 lifestyle questionnaire (Table 7). 

There were no significant differences between groups or between baseline and week-12 values from the Perceived Stress Scale (PSS) questionnaire results (Table 8).

The Pittsburgh Sleep Quality Index (PSQI-10) questionnaire results showed that there were significant differences between groups at baseline (Table 9). At baseline, sleep efficiency scores were significantly lower in the placebo group compared to the PEA group (*p* = 0.04). 

## 4. Discussion

Chronic low-grade inflammation is a significant contributor to the development and progression of chronic degenerative diseases, often arising from prolonged exposure to stress and poor dietary choices [39,40]. In this context, including dietary supplementation as part of a balanced diet offers a valid approach to mitigating the risk and severity of these diseases [5]. PEA is a well-tolerated dietary supplement with anti-inflammatory, analgesic, antimicrobial, immunomodulatory, and neuroprotective properties [41]. PEA’s anti-inflammatory properties and ability to modulate the LPS pathway suggest that PEA may exert its anti-inflammatory action, in part, by influencing the gut microbiome [19,42]. This study aimed to examine the potential of PEA to modify the gut microbiome and the subsequent effects on health markers compared to a placebo in overweight but otherwise healthy adults aged 18–65 years old.

The findings of our study indicate that the active treatment group (PEA) had significant reductions in triglyceride and IL-2 levels after 12 weeks of supplementation. This is consistent with previous studies demonstrating the potential role of PEA in modulating lipid metabolism and immune responses [43,44]. For instance, the increase in hepatic triglycerides in high-fat diet mice was reduced by eight weeks of ultramicronised PEA (1.5%) treatment [43]. In a double-blind, randomised, placebo-controlled trial involving adults recently diagnosed with COVID-19, four weeks of PEA supplementation led to significant reductions in IL-1β and IL-2 concentrations [44]. 

The overall microbiota of both the placebo and PEA groups, before and after the intervention, exhibited a composition typically observed in the gut microbiota of the human body. This composition consisted of Bacteroidetes, Firmicutes, Actinomycetes, Fusobacteria, and Verrucomicrobia, with Bacteroidetes and Firmicutes being the dominant phyla [45]. At the species level, the dominant species are either *Faecalibacterium prausnitzii* or *Prevotella copri* or *Bacteroides vulgatus* or *Bacteroides uniformis* or *Alistipes putredinis* or *Bacteroides stercoris* or *Bacteroides dorei*. Most of these species are involved in the breakdown of complex carbohydrates and contribute to the production of short-chain fatty acids and other metabolites [46,47,48]. 

Although the overall microbiota profiles remained largely stable between baseline and final measurements based on the multivariate analysis, univariate analysis revealed significant differences in the relative abundances of various microbial species. Interestingly, none of the differentially abundant species were among the dominant species in the top 20. This suggests that while the dominant species remained relatively consistent, there were noteworthy shifts in the less-abundant microbial members. 

The presence of comparable top 20 metabolic pathways in both groups indicates a significant level of shared characteristics or resemblance in the metabolic profiles of these two groups. Moreover, although the overall functional profiles (quantitative abundance) of the microbiomes in both groups did not change significantly, there were changes in the specific functional content of the gut microbiome functions in the PEA group. This could indicate that certain functions within the gut microbiome were influenced by factors related to the PEA intervention, whereas such changes were not observed in the placebo supplementation group.

Interestingly, the PEA group demonstrated a reduction in the activity of several metabolic pathways after 12 weeks of supplementation. For instance, the pathways involved in the degradation of various aromatic compounds, interconversion of NAD^+^/NADH and NADP^+^/NADPH to maintain cellular redox balance, L-glutamate degradation IV, and L-glutamate degradation VIII (propanoate) were affected. The reduction in the activity of the aromatic compound degradation pathway could impact the microorganism’s ability to break down aromatic compounds, potentially affecting energy production and biosynthesis [49]. The alterations in NAD^+^ and NADP^+^ interconversion pathways may disrupt the cell’s redox state in the intestinal lumen, which is crucial for a variety of cellular processes including energy generation and mitochondrial function [50]. The L-glutamate degradation IV pathway, also known as the GABA (γ-aminobutyric acid) shunt pathway, is an essential metabolic pathway involved in the conversion of the amino acid glutamate into the neurotransmitter GABA [51,52]. The L-glutamate degradation VIII (propanoate) pathway involves a series of enzymatic reactions that convert L-glutamate into propanoate (propionate) [53,54]. Overall, these metabolic pathways facilitate the degradation of glutamate, thereby regulating neurotransmission, maintaining neuronal balance, and contributing to various neurological functions [55,56]. It is worth noting that PEA interacts with the endocannabinoid system, particularly cannabinoid receptors like CB1 and CB2 [41]. Given the endocannabinoid system’s role in modulating neurotransmitter release, this interaction may potentially influence glutamate metabolism and degradation [57].

The NAD^+^/NADH and NADP^+^/NADPH interconversion pathway regulates cellular metabolism and maintains redox balance while aiding in detoxification and optimising resource allocation for critical cellular functions, such as growth, DNA replication, and responses to environmental stressors [50,58]. This is advantageous for redirecting metabolic resources when needed and defending against harmful substances [59]. Downregulation of these pathways may influence triglyceride levels by indirectly affecting the metabolic processes and nutrient availability essential for triglyceride synthesis and metabolism [60]. However, the specific effects would depend on the extent of pathway disruption and the broader metabolic context. The potential reductions in triglycerides via the NAD^+^/NADH and NADP^+^/NADPH interconversion pathway requires further investigation.

However, as all plasma markers (i.e., inflammation, oxidative damage, intestinal permeability, endotoxins, and general health) in the PEA group either remained stable or improved, it is unlikely that the reduction in the activity of these microbiome driven pathways were affected negatively. Rather, it may be that PEA supplementation was able to have a beneficial effect, which allows these pathways to be downregulated yet maintain a healthy state.

Conversely, 12 weeks of PEA supplementation resulted in elevated levels of the molybdopterin biosynthesis pathway and the dTDP-glucose-derived O-antigen building blocks biosynthesis pathway. Molybdopterin is a cofactor essential for the function of molybdenum-containing enzymes. These enzymatic proteins play a pivotal role in a wide spectrum of crucial metabolic reactions within cells [61]. O-antigens are components of bacterial LPS and are essential for bacterial cell surface recognition and immune interactions [62]. 

Elevated levels of the molybdopterin biosynthesis pathway are associated with the functioning of various enzymes, which can impact the immune responses [63]. The modulation of this pathway may influence the production or activity of immune-related molecules, such as IL-2, which plays a pivotal role in regulating the immune system [64].

Similarly, the dTDP-glucose-derived O-antigen building blocks biosynthesis pathway, by influencing carbohydrate biosynthesis, can potentially impact immune factors like IL-2 by regulating T-cell activation and proliferation [65,66]. This aligns with PEA’s known mechanism of action, which involves reducing the activation of immune cells [41]. Consequently, the observed decrease in IL-2 levels in individuals supplemented with PEA may be attributed to these immunomodulatory effects, offering a promising direction for further investigation into the therapeutic potential of PEA in immune-related disorders. 

O-antigen molecules are polysaccharides protruding from LPS, and O-antigen presence determines if the LPS is classified as rough or smooth. More O-chains would make the LPS smooth, whereas the lack of, or reduction in, O-chains would result in rough LPS. Stranahan and Arenas-Gamboa [67] reported that a rough phenotype indicates pathogenic stealth and persistence. Increased activity in this pathway, as shown in the PEA group, would result in smooth LPS and reduce its pathogenicity. As expected, the increase in dTDP-glucose-derived O-antigen building blocks in the PEA group did not lead to a corresponding change in total LPS levels; rather, it is expected to alter the type of LPS produced. This could be due to regulatory mechanisms controlling LPS production, alternative biosynthetic pathways, or other potential limiting factors within the LPS biosynthesis pathway [68]. Therefore, the upregulation of dTDP-glucose derived O-antigen building blocks biosynthesis pathways in the absence of a decrease in LPS may still exert beneficial effects by reducing LPS pathogenesis. However, further investigation is required to better understand the connection between LPS and PEA.

Together these results show that despite the changes to the metabolic pathways, the overall health and wellbeing of the participants did not change. This is evident by the liver function (fibroscan and biochemistry), general health (24 h dietary recall, SF-36, PSS, the PSQI-10), and faecal SCFAs and calprotectin not changing throughout the 12 weeks. 

Despite the positive findings of this study, there were a number of limitations and improvements that could be made in subsequent investigations of this nature. First, the number of participants that were able to provide blood was reduced due to difficulties attending the clinic due to COVID-19. Therefore, the data that we were able to obtain from biochemistry may have been limited. Had all subjects been able to have their blood collected, this may have allowed for more changes to be detected. Another limitation was the number of participants who provided faecal samples that were not collected correctly. Despite the detailed instructions, participants returned a number of samples that were either insufficient in volume or they had removed the preservation solution. This meant that these samples had to be removed from the analysis. To help reduce the impact of both the biochemistry and faecal sample limitations, additional participants may be required to allow for a greater dropout rate than was allowed for here.

## 5. Conclusions

Overall, this study showed significant reductions in triglyceride and IL-2 levels and changes to the microbiome function after 12 weeks of PEA supplementation. While the overall composition of gut microbiota remained stable, specific functional changes were observed in the PEA group, suggesting a potential modulation of metabolic pathways. PEA supplementation resulted in the downregulation of pathways involved in aromatic compound degradation, NAD^+^/NADH and NADP^+^/NADPH interconversion, and L-glutamate degradation. Conversely, pathways related to molybdopterin and O-antigen biosynthesis were upregulated. These changes imply the existence of potential immunomodulatory effects of PEA, influencing immune responses and metabolic processes.

To our knowledge, this study is the first to investigate the specific microbiome functional changes following PEA supplementation. The significance of these findings lies in the potential therapeutic application of PEA. Alterations in microbiome function have been associated with various conditions, and understanding how an intervention affects the microbiome may provide insights into disease mechanisms and potential therapeutic strategies for various health conditions. Future studies incorporating PEA therapy could explore conditions known to be influenced by microbiome function, thereby enhancing our understanding of PEA’s effects on both microbiome function and disease management.

This study has initiated exploration into the potential effects of PEA on the microbiome and metabolic pathways. However, several changes could enhance future investigations. The dosage employed in this study aligns with historical data indicating efficacy. However, employing a higher dosage might yield a more pronounced impact during the 12-week supplementation period utilised here. Alternatively, conducting a longer-term study could facilitate more substantial metabolic alterations. The duration of 12 weeks may have been insufficient to elicit significant microbiome changes capable of influencing metabolic processes and overall health parameters, such as biochemistry.

## Figures and Tables

**Table 1:** Baseline demographic and anthropometric measurements.

Parameter	Group A (PEA; *n* = 29)		Group B (Placebo; *n* = 15)	
	Baseline	Week 12	Baseline	Week 12
Age (years)	49.8 (10.7)	-	47.3 (10.21)	-
Height (m)	1.69 (0.09)	1.69 (0.08)	1.73 (0.07)	1.73 (0.07)
Weight (kg)	99.29 (14.28)	97.43 (13.94)	103.78 (15.1)	99.17 (14.56)
BMI (kg/m^2^)	34.37 (2.65)	34.06 (2.66)	34.48 (3.49)	33.2 (3.45)
Waist circumference (cm)	105.02 (9.74)	103.4 (10.56)	105.77 (11.4)	104.5 (11.98)
Hip circumference (cm)	119.77 (8.72)	119.1 (8.86)	118.86 (7.85)	114.91 (5.22)
Systolic BP (mmHg)	131.43 (13.56)	128.86 (14.09)	131.7 (13.08)	130 (13.49)
Diastolic BP (mmHg)	88.78 (9.31)	84.66 (7.97)	85.7 (7.23)	85.8 (7.72)
Pulse (BPM)	71.59 (12.13)	70.66 (8.44)	68.7 (11)	67.27 (11.12)

Data is shown as mean (SD).

**Table 2:** Twenty-four hour diet recall data.

Parameter	Group A (PEA; *n* = 29)				Group B (Placebo; *n* = 15)			
	Baseline	Week 6	Week 12	Change	Baseline	Week 6	Week 12	Change
Total intake (kJ)	7739.6 (2782.7)	8822.4 (3444.1)	8165.8 (2589.8)	426.2 (2917.7)	8739.7 (3766.2)	9362.1 (4033.8)	8507.9 (2185.7)	−231.7 (4319.1)
Fat (g)	84.3 (35.4)	94.8 (48.7)	88.4 (34.1)	4.1 (34.6)	86.7 (41.1)	100.7 (47.6)	90.9 (29.5)	4.3 (51.8)
Carbohydrate (g)	176.6 (95.5)	195.3 (90.8)	173.3 (86.5)	−3.3 (83.1)	199.9 (117.0)	205.8 (112.3)	188.1 (64.9)	−11.8 (130.4)
Protein (g)	80.7 (31.2)	100.7 (40.2)	94.5 (34.4)	13.8 (46.7)	103.7 (68.7)	106.4 (47.5)	97.7 (30.3)	−6.0 (74.0)
Fiber (g)	19.9 (8.3)	21.9 (10.6)	19.5 (9.0)	−0.4 (10.2)	21.4 (19.1)	18.9 (5.4)	20.8 (9.7)	−0.6 (24.4)

Data shown as mean (SD).

**Table 3:** Biochemical marker outcomes.

Parameter	Group A (PEA; *n* = 27)				Group B (Placebo; *n* = 13)			
	Baseline	Week 6	Week 12	Change	Baseline	Week 6	Week 12	Change
FABP2	4.68 (7.05)	4.94 (7.23)	4.64 (7.02)	−0.04 (0.82)	4.99 (7.54)	4.62 (7.39)	4.45 (7.04)	−0.54 (1.50)
TGF-β1	105.01 (181.77)	77.89 (150.09)	62.16 (122.41) ^b^	−42.85 (86.71)	84.98 (75.53)	35.74 (77.81)	66.74 (110.31)	−18.23 (111.47)
GST	1.07 (0.91)	1.09 (0.87)	1.14 (1.04)	0.08 (0.45)	1.04 (1.02)	1.01 (0.83)	0.9 (0.8)	−0.14 (0.42)
GLP−1	48.43 (43.45)	52.26 (43.5)	49.45 (44.61)	1.02 (7.05)	32.66 (32.6)	31.82 (35.48)	31.04 (32.9)	−1.62 (4.92)
Zonulin	1.81 (1.06)	1.75 (1.06)	1.75 (1.14)	−0.05 (1.07)	2.44 (1.38)	1.98 (1.36)	1.92 (1.35)	−0.52 (0.78)
MCP-2	63.09 (71.15)	58.41 (72.1)	56.86 (67.53)	−6.23 (29.64)	70.76 (69.22)	58.06 (57.35)	64.69 (67.99)	−6.07 (25.13)
Occludin	71.43 (56.48)	54.98 (43.64)	60.96 (40.91)	−10.47 (31.72)	84.1 (59.24)	76.12 (55.26)	70.95 (59.08)	−13.15 (27.36)
Mucin2	0.02 (0.08)	0.04 (0.16)	0.03 (0.11)	0.01 (0.12)	0.75 (1.38)	0.72 (2.47)	0.67 (1.93)	−0.09 (1.91)
LPS	5.63 (1.94)	5.64 (2.15)	4.9 (1.3) ^b^	−0.73 (1.52)	5.84 (1.87)	5.25 (1.6)	5.52 (2.15)	−0.32 (2.21)
Albumin (g/L)	45.47 (1.9)	-	45.45 (2.02)	−0.02 (1.52)	45.56 (1.89)	-	45.73 (2.38)	0.18 (1.31)
ALP (U/L)	67.56 (20.57)	-	66.84 (22.42)	−0.72 (13.95)	62.83 (20.31)	-	66.84 (22.49)	4.33 (12.30)
ALT(U/L)	29.03 (14.25)	-	29.61 (16.76)	0.58 (13.57)	20.28 (9.41) *	-	19.8 (10.57) *	−0.48 (8.05)
AST (U/L)	29.04 (12.37)	-	30.92 (11.15)	1.88 (17.54)	26.2 (10.52)	-	21.93 (8.22) *	−4.27 (8.73)
CHO (mmol/L)	5.45 (1.12)	-	5.52 (1.05)	0.07 (0.46)	5.56 (0.81)	-	5.63 (0.95)	0.07 (0.53)
Homocysteine (umol/L)	7.79 (1.84)	-	7.72 (1.76)	−0.07 (2.17)	7.76 (3.28)	-	7.46 (3.06)	−0.30 (1.55)
HDL-C (mmol/L)	1.46 (0.51)	-	1.49 (0.51)	0.03 (0.28)	1.39 (0.53)	-	1.55 (0.61)	0.16 (0.28)
LDL-C (mmol/L)	3.37 (0.89)	-	3.5 (0.89)	0.13 (0.37)	3.46 (0.8)	-	3.58 (1.01)	0.12 (0.74)
TG (mmol/L)	1.63 (1.0)	-	1.45 (0.77)	−0.18 (0.47)	1.33 (0.42)	-	1.4 (0.52)	0.07 (0.18) *
Total protein (g/L)	69.1 (3.9)	-	68.72 (4.06)	−0.38 (2.54)	67.28 (4.09)	-	67.88 (3.9)	0.56 (1.69)
GGT (U/L)	28.28 (17.8)	-	30.2 (16.68)	1.92 (10.35)	29.5 (21.69)	-	31.42 (21.69)	1.92 (9.40)
Tbil (umol/L)	6.13 (3.91)	-	6.55 (4.95)	0.42 (4.19)	10.25 (9.73)	-	12.78 (8.81) *	2.53 (6.86)
Creatinine (umol/L)	86.6 (16.71)	-	81.55 (13.53)	−5.06 (13.35)	93.75 (17.7)	-	88.39 (18.05)	−5.37 (11.32)
Glucose (mmol/L)	5.54 (1.25)	-	5.26 (1.26)	−0.28 (1.07)	5.16 (0.73)	-	5.02 (0.79)	−0.14 (0.89)
hsCRP (mg/L)	3.54 (3.42)	-	2.92 (2.24)	−0.61 (2.94)	5.1 (4.7)	-	5.72 (5.28)	0.63 (4.98)
IFN-γ (pg/mL)	41.1 (27.32)	42.65 (25.56)	41.98 (28.75)	0.88 (6.11)	32.56 (32.4)	23.83 (15.15) *	36.84 (36.13)	4.28 (6.85)
IL1-β (pg/mL)	2.60 (2.17)	2.82 (2.21)	2.54 (1.37)	−0.05 (1.00)	1.56 (0.43)	1.40 (0.43) *	1.60 (0.56) *	−0.02 (0.18)
IL-2 (pg/mL)	5.44 (5.2)	5.53 (5.29)	5.2 (4.41)	−0.24 (1.06)	4.16 (4.05)	3.03 (1.26) *	4.86 (5.13)	0.70 (1.38) *
TNF-α (pg/mL)	12.56 (4.49)	12.63 (4.52)	12.41 (4.45)	−0.15 (1.88)	13.93 (3.83)	12.73 (4.11)	14.51 (4.91)	0.58 (2.23)
RBC Glutathione (uM)	1565.4 (330.3)	1574.5 (385.7)	1613.7 (369.5)	48.3 (228.4)	1648.3 (441.5)	1754.3 (489.8)	1716.4 (538.6)	68.1 (161.6)
Plasma Glutathione (uM)	7.54 (3.56)	6.89 (2.46)	6.64 (2.91)	−0.90 (3.00)	6.56 (1.41)	6.28 (1.57)	6.77 (1.46)	0.20 (2.15)

Data shown as mean (SD); * indicates a significant difference between groups (*p* < 0.05); ^b^ indicates a significant difference within group (*p* < 0.05). ALP, alkaline phosphatase; ALT, alanine transaminase; AST, aspartate aminotransferase; CHO, cholesterol; FABP2, fatty acid binding protein 2; GGT, gamma-glutamyl transferase; GLP-1, glucagon-like peptide 1; GST, glutathione transferase; HDL-C, high density lipoprotein cholesterol; hsCRP, high-sensitivity C-reactive protein; IFN-γ, interferon gamma; IL1-β, interleukin 1 beta; IL-2, interleukin 2; LDL-C, low density lipoprotein cholesterol; LPS, lipopolysaccharide; MCP-2, monocyte chemoattractant protein 2; RBC, red blood cell; Tbil, total bilirubin; TG, triglycerides; TGF-β1, transforming growth factor beta 1; TNF-α, tumour necrosis factor alpha.

**Table 4:** Differential pathways in the PEA group (Group A) were identified with Metastat paired analysis.

Pathways	Higher at
PROTOCATECHUATE-ORTHO-CLEAVAGE-PWY: protocatechuate degradation II ortho cleavage pathway	Baseline
PWY-7245: superpathway of NAD/NADP NADH/NADPH interconversion yeast	Baseline
PWY-5417: catechol degradation III ortho cleavage pathway	Baseline
PWY-5431: aromatic compounds degradation via beta ketoadipate	Baseline
PWY-7165: L ascorbate biosynthesis VIII engineered pathway	Baseline
PWY-5181: toluene degradation III aerobic via p cresol	Baseline
CATECHOL ORTHO CLEAVAGE PWY: catechol degradation to beta ketoadipate	Baseline
PWY-6182: superpathway of salicylate degradation	Baseline
PWY-7268: cytosolic NADPH production yeast	Baseline
PWY-6185: 4 methylcatechol degradation ortho cleavage	Baseline
PWY-7317: superpathway of dTDP glucose derived O antigen building blocks biosynthesis	Final
PWY-6823: molybdopterin biosynthesis	Final

All pathways were significantly altered (*p* < 0.001, FDR < 0.05).

**Table 5:** SCFA and calprotectin measure outcomes.

	Group A (PEA; *n* = 19)		Group B (Placebo; *n* = 12)	
	Baseline	Week 12	Baseline	Week 12
(umol SCFA/g dry weight)				
Acetic acid	94.17 (53.69)	97.57 (73.47)	117.28 (73.33)	77.76 (32.11)
Propionic acid	22.95 (17.19)	23.08 (17.47)	26.71 (21.42)	17.35 (11)
Isobutyric acid	1.92 (1.38)	2.07 (1.63)	1.75 (1.06)	1.80 (1.25)
Butyric acid	16.30 (12.93)	19.22 (15.89)	21.12 (13.54)	14.72 (7.19)
Isovaleric acid	2.67 (2.07)	2.94 (2.48)	2.24 (1.54)	2.58 (1.92)
Valeric acid	2.33 (1.71)	2.34 (1.9)	2.94 (2.02)	2.19 (1.25)
Hexanoic acid	0.61 (1.05)	0.59 (0.69)	0.86 (1.24)	0.96 (1.5)
Heptanoic acid	92.99 (54.42)	94.23 (45.04)	89.08 (48.02)	82.65 (42.04)
Calprotectin (ug/g)	195.32 (738.13)	243.98 (950.19)	226.65 (854.68)	304.57 (1167.96)
(% SCFA)				
Acetic acid	40.06 (9.6)	38.6 (12.07)	43.30 (12.22)	39.1 (13.29)
Propionic acid	9.19 (3.26)	8.88 (3.25)	9.55 (3.94)	8.37 (4.05)
Isobutyric acid	0.88 (0.51)	0.84 (0.48)	0.81 (0.56)	0.92 (0.59)
Butyric acid	6.57 (3.29)	7.26 (3.66)	7.63 (2.1)	7.38 (3.15)
Isovaleric acid	1.26 (0.81)	1.18 (0.75)	1.10 (0.9)	1.34 (0.95)
Valeric acid	1.01 (0.5)	0.91 (0.47)	1.15 (0.49)	1.10 (0.56)
Hexanoic acid	0.26 (0.37)	0.25 (0.29)	0.32 (0.4)	0.53 (0.75)
Heptanoic acid	40.76 (14.47)	42.08 (16.98)	36.14 (17.18)	41.25 (20.55)

Data shown as mean (SD).

**Table 6:** Liver scan results.

	Group A (PEA; *n* = 29)		Group B (Placebo; *n* = 15)	
	Baseline	Week 12	Baseline	Week 12
Liver size (mm)	134.23 (39.16)	128.63 (27.59)	141.94 (21)	132.89 (25.3)
Portal vein measure (mm)	11.47 (1.98)	12.38 (1.66)	11.71 (2.61)	12.33 (2.58)
Hepatopetal flow with doppler velocity (cm/s)	19.89 (4.61)	22.02 (7.81)	17.56 (3.91)	18.85 (4.99)
Median liver stiffness value (kPa)	4.87 (1.18)	5.05 (1.46)	5.03 (1.02)	5.65 (1.31)
Median velocity (m/s)	1.28 (0.17)	1.45 (1.0)	1.28 (0.13)	1.71 (1.48)

Data shown as mean (SD).

**Table 7:** SF-36 questionnaire outcomes.

	Group A (PEA; *n* = 29)			Group B (Placebo; *n* = 15)		
	Baseline	Week 6	Week 12	Baseline	Week 6	Week 12
Physical functioning	81.48 (18.65)	82.59 (15.59)	85.56 (13.03)	90 (14.94)	93.57 (7.70)	92.14 (8.93)
Role limitations due to physical health	75.93 (31.38)	74.07 (36.35)	75.93 (37.65)	98.21 (6.68)	96.43 (9.08)	87.5 (32.15)
Role limitations due to emotional problems	75.31 (35.32)	69.14 (39.14)	66.67 (42.37)	85.71 (36.31)	90.48 (20.37)	90.48 (27.51)
Energy/fatigue	53.7 (19.93)	55.74 (20.79)	57.04 (16.94)	67.5 (19.88)	71.79 (15.27)	68.93 (16.19)
Emotional well-being	75.41 (16.14)	77.48 (12.56)	74.07 (16.28)	76 (12.94)	81.71 (7.64)	80.86 (8.76)
Social functioning	84.26 (20.09)	88.43 (14.68)	85.19 (19)	90.18 (18.46)	97.32 (5.32)	94.64 (11.72)
Pain	77.13 (18.93)	78.7 (14.13)	74.44 (16.47)	86.79 (9.88)	91.25 (14.63)	90.36 (12.12)
General health	57.22 (21.85)	59.07 (20.34)	56.67 (20.62)	73.57 (16.81)	76.07 (13.61)	76.07 (13.18)

Data shown as mean (SD).

**Table 8:** PSS questionnaire outcomes.

	Group A (PEA; *n* = 29)			Group B (Placebo; *n* = 15)		
	Baseline	Week 6	Week 12	Baseline	Week 6	Week 12
Total scores	11.24 (6.08)	11.23 (5.83)	12.03 (7.23)	14.05 (5.51)	9.53 (5.11)	9.67 (4.37)

Data shown as mean (SD).

**Table 9:** PSQI-10 questionnaire outcome.

	Group A (PEA; *n* = 29)			Group B (Placebo; *n* = 15)		
	Baseline	Week 6	Week 12	Baseline	Week 6	Week 12
Total score	11.52 (5.59)	12.18 (6.86)	12.34 (5.56)	8.53 (4.32)	7.00 (4.35) *	7.73 (3.75) *
Subjective sleep quality	1.07 (0.7)	1.00 (0.61)	0.97 (0.73)	0.87 (0.52)	0.79 (0.58)	0.87 (0.52)
Sleep latency	1.93 (1.83)	1.43 (1.67)	1.83 (1.79)	1.13 (1.25)	0.71 (1.14)	1.00 (1.25)
Sleep duration	0.34 (0.55)	0.50 (0.64)	0.45 (0.57)	0.27 (0.46)	0.14 (0.36)	0.13 (0.35)
Hours in bed	9.54 (3.97)	9.20 (3.43)	9.01 (4.77)	7.88 (0.94)	8.26 (0.77)	8.21 (0.81)
Sleep efficiency	79.82 (19.23)	80.85 (20.1)	79.99 (20.32)	91.03 (10.48) *	90.60 (9.2)	83.03 (23.85)
Component: Sleep efficiency	0.86 (1.03)	0.93 (1.09)	1.07 (1.11)	0.33 (0.62)	0.29 (0.47) *	0.40 (0.83)
Sum Qs 5B-5J	7.90 (4.4)	8.75 (5.5)	8.69 (4.42)	5.87 (3.93)	5.07 (3.27) *	5.33 (3.48) *
Sleep disturbance	1.24 (0.58)	1.36 (0.68)	1.38 (0.49)	1.13 (0.52)	0.93 (0.47) *	1.07 (0.46)
Use of sleep medication	0.24 (0.51)	0.32 (0.72)	0.31 (0.71)	0.40 (1.06)	0.21 (0.8)	0.20 (0.77)
Qs 8–9	1.17 (1.04)	1.25 (1.0)	1.28 (1.13)	0.67 (0.72)	0.50 (0.76) *	0.67 (0.62)
Daytime dysfunction	0.83 (0.66)	0.89 (0.57)	0.83 (0.6)	0.53 (0.52)	0.36 (0.5) *	0.60 (0.51)
Global score	6.52 (3.93)	6.43 (3.41)	6.78 (3.95)	4.67 (2.99)	3.43 (2.44) *	4.27 (2.79) *

Data shown as mean (SD); * indicates a significant difference between groups (*p* < 0.05).

## Data Availability

The datasets generated and/or analysed during the current study are not publicly available due to commercial interests but are available from the corresponding author on reasonable request.

## Supplementary Materials

The following supporting information can be downloaded at: https://www.mdpi.com/article/10.3390/biomedicines12071620/s1, Table S1: Differential species in the PEA group (Group A) identified with Metastat paired analysis. Table S2: Differential species in the placebo group (Group B) identified with Metastat paired analysis.
