# Supplementary material for: Effect of Palmitoylethanolamide Compared to a Placebo on the Gut Microbiome and Biochemistry in an Overweight Adult Population: A Randomised, Placebo Controlled, Double-Blind Study
Source: Biomedicines. 2024 Jul 20;12(7):1620. doi: 10.3390/biomedicines12071620 (PMC11274356; doi:10.3390/biomedicines12071620)
Supplement: Supplementary file 1 [file biomedicines-12-01620-s001.zip › Supplementary Table S1.pdf]

Supplementary Table S1: Differential species in the PEA group (Group A) identified with Metastat paired analysis.

All species were significantly different (*P* < 0.001, FDR < 0.01)

Species	Higher in
Methanobrevibacter smithii	Baseline
Haemophilus sp HMSC71H05	Baseline
Prevotella corporis	Baseline
Mitsuokella jalaludinii	Baseline
Jeotgalicoccus halotolerans	Baseline
Prevotella timonensis	Baseline
Citrobacter werkmanii	Baseline
Christensenella minuta	Baseline
Porphyromonas sp HMSC065F10	Baseline
Flavonifractor sp An100	Baseline
Lachnoclostridium sp An131	Baseline
Lachnoclostridium sp An138	Baseline
Parabacteroides gordonii	Baseline
Bacteroides sp D2	Baseline
Propionibacterium freudenreichii	Baseline
Actinomyces sp ICM47	Baseline
Lactococcus lactis	Baseline
Lactobacillus acidophilus	Baseline
Eubacteriaceae bacterium CHKCI005	Baseline
Paraprevotella xylaniphila	Final
Prevotella sp CAG 5226	Final
Prevotella stercorea	Final
Prevotella sp AM42 24	Final
Clostridium neonatale	Final
Blautia sp N6H1 15	Final
Bacteroides coprophilus	Final
Faecalicoccus pleomorphus	Final
Sellimonas intestinalis	Final
Erysipelothrix larvae	Final
Bifidobacterium dentium	Final
Proteus mirabilis	Final
Ruminococcus obeum CAG 39	Final
Lactobacillus plantarum	Final
Oxalobacter formigenes	Final
Streptococcus gordonii	Final
Saccharomyces cerevisiae	Final
Blautia producta	Final
Citrobacter portucalensis	Final
Blautia coccoides	Final
Lactobacillus salivarius	Final
Eubacterium limosum	Final

Veillonella tobetsuensis	Final
Streptococcus mitis	Final
Clostridium sp chh4 2	Final
Romboutsia ilealis	Final
Veillonella rogosae	Final
Prevotella buccalis	Final
