# Supplementary material for: Effect of Palmitoylethanolamide Compared to a Placebo on the Gut Microbiome and Biochemistry in an Overweight Adult Population: A Randomised, Placebo Controlled, Double-Blind Study
Source: Biomedicines. 2024 Jul 20;12(7):1620. doi: 10.3390/biomedicines12071620 (PMC11274356; doi:10.3390/biomedicines12071620)
Supplement: Supplementary file 1 [file biomedicines-12-01620-s001.zip › Supplementary Table S2.pdf]

Supplementary Table S2: Differential species in the placebo group (Group B) identified with Metastat paired analysis. All species were significant with $P < 0.001$ and $FDR < 0.01$

Species	Higher in
Prevotella sp CAG 891	Baseline
Bifidobacterium pseudolongum	Baseline
Turicibacter sanguinis	Baseline
Megamonas funiformis	Baseline
Erysipelatoclostridium ramosum	Baseline
Mitsuokella multacida	Baseline
Clostridium innocuum	Baseline
Bifidobacterium animalis	Baseline
Butyricicoccus pullicaecorum	Baseline
Enterobacter mori	Baseline
Clostridium disporicum	Baseline
Veillonella atypica	Baseline
Methanosphaera stadtmanae	Baseline
Helicobacter bilis	Baseline
Fusobacterium mortiferum	Baseline
Candidatus Gastranaerophilales bacterium	Baseline
Bifidobacterium catenulatum	Baseline
Streptococcus lutetiensis	Baseline
Enterorhabdus caecimuris	Baseline
Streptococcus sp A12	Baseline
Saccharomyces cerevisiae	Baseline
Veillonella infantium	Baseline
Clostridiales bacterium 1 7 47FAA	Baseline
Actinomyces sp ICM47	Baseline
Alistipes onderdonkii	Final
Brachyspira sp CAG 700	Final
Prevotella corporis	Final
Clostridium bolteae CAG 59	Final
Parabacteroides sp CAG 409	Final
Desulfovibrio fairfieldensis	Final
Cloacibacillus evryensis	Final
Pseudomonas aeruginosa group	Final
Brachyspira pilosicoli	Final
Bacteroides sp CAG 530	Final
Citrobacter amalonaticus	Final
Atopobium minutum	Final
Prevotella timonensis	Final
Prevotella bivia	Final
Bifidobacterium dentium	Final
Prevotella disiens	Final
Christensenella minuta	Final

Ruminococcus obeum CAG 39	Final
Catabacter hongkongensis	Final
Parabacteroides gordonii	Final
Actinomyces turicensis	Final
Gardnerella vaginalis	Final
Butyribacterium methylotrophicum	Final
Porphyromonas asaccharolytica	Final
